# Towards light-coupled sample preparation for time-resolved cryoEM studies

**DOI:** 10.1107/S2052252526005324

**Published:** 2026-06-29

**Authors:** Kyprianos Hadjidemetriou, Sofia Jaho, Pierre Aller, Zhanru Yu, Benedikt M. Kessler, Stephen P. Muench, Nikil Kapur, Gabriel Karras, Robin L. Owen, Peijun Zhang

**Affiliations:** a Diamond Light Source, Harwell Science and Innovation Campus, DidcotOX11 0DE, United Kingdom; b Research Complex at Harwell Rutherford Appleton Laboratory DidcotOX11 0FA United Kingdom; c Target Discovery Institute, Centre for Medicines Discovery, Nuffield Department of Medicine University of Oxford Roosevelt Drive OxfordOX3 7FZ United Kingdom; d Chinese Academy of Medical Sciences Oxford Institute, Nuffield Department of Medicine University of Oxford Roosevelt Drive OxfordOX3 7FN United Kingdom; e School of Biomedical Sciences, Faculty of Biological Sciences University of Leeds LeedsLS2 9JT United Kingdom; f Astbury Centre for Structural and Molecular Biology University of Leeds LeedsLS2 9JT United Kingdom; g School of Mechanical Engineering University of Leeds LeedsLS2 9JT United Kingdom; h Division of Structural Biology, Nuffield Department of Medicine University of Oxford OxfordOX3 7BN United Kingdom; MRC Laboratory of Molecular Biology, United Kingdom

**Keywords:** cryo-electron microscopy, cryoEM, cryo-electron tomography, cryoET, time-resolved studies, photocages, bacterial chemotaxis, minicells, on-grid spectroscopy, advances in microscope hardware, imaging, multi-protein complexes

## Abstract

The dynamic nature of protein and macromolecular complexes means that the capture of multiple sequential states along a reaction pathway can provide much greater insight into function than that obtained from a single static structure. We present a set of modular, easy-to-implement tools and workflows for optical excitation, on-grid characterization and tightly coupled rapid vitrification, establishing a proof-of-principle framework for time-resolved cryoEM and cryo-electron tomography (cryoET). We apply this framework to *E. coli* chemotaxis, in which serine-sensitive chemoreceptors initiate signalling upon ligand binding and undergo critical conformational changes within the chemosensory arrays. Using DMNB-caged serine [*O*-(4,5-di­meth­oxy-2-nitro­benzyl)-l-serine] as a model trigger, we quantified its photophysical properties and uncaging efficiency using UV–Vis spectroscopy and two-dimensional gas chromatography mass spectrometry (GC×GC-MS). Coupling a femtosecond-pulsed laser to a Vitrobot enabled reproducible reaction-to-vitrification delays of ∼150 ms, yielding intact *E. coli* minicells with well-preserved chemotaxis arrays suitable for *in situ* structural analysis by cryoET. This integrated approach provides a robust and generalisable framework for millisecond time-resolved cryoET, laying the groundwork for capturing transient conformational states in their native cellular context.

## Abstract 2

We present an integrated sample preparation workflow that combines photocaged ligand activation and controlled triggering, coupled with rapid vitrification to enable time-resolved cryoET studies on bacterial chemotaxis at millisecond timescales.

## 1. Introduction

Protein and macromolecular complexes are perpetually dynamic and undergo conformational changes when they are involved in a chemical or biological process, respond to stimuli or interact with each other. The scale and the magnitude of the structural changes are closely coupled to the timescale of corresponding biological processes. These range from femtosecond bond vibrations to enzymatic turnover on timescales of milliseconds to seconds and even minutes (Banari *et al.*, 2025 ▸; Mäeots & Enchev, 2022 ▸). Capturing a set of intermediate states, rather than a single static structure, is the goal of time-resolved structural biology, providing insights into the reaction mechanism of the system of interest.

Several approaches have been suggested for ‘pausing’ reactions in time or preferentially increasing the population of certain intermediate states, including inhibitor binding (Westmoreland *et al.*, 2024 ▸; Zhou *et al.*, 2022 ▸), active-site mutations (Oda *et al.*, 2020 ▸; Mulelu *et al.*, 2019 ▸), pH (Yue *et al.*, 2025 ▸; Rovšnik *et al.*, 2021 ▸) and temperature variation (Chen *et al.*, 2019 ▸), that can then be characterized by techniques such as macromolecular cryo X-ray crystallography (cryoMX) or cryo-electron microscopy (cryoEM) (Tsai *et al.*, 2022 ▸; Mäeots & Enchev, 2022 ▸). While these approaches have provided important functional insights for many biological systems, their applicability is highly system-dependent and they can sometimes yield structures that deviate substantially from the native state and environment of the samples (Tsai *et al.*, 2022 ▸; Banari *et al.*, 2025 ▸).

A significant application of time-resolved structural biology is that it allows the characterization of short-lived intermediate states by initiating a reaction and capturing structural snapshots at defined time points (Banari *et al.*, 2025 ▸). This is achieved through relatively fast physical, chemical and biological triggering, such as light activation, rapid mixing, temperature or pH jumps. Time-resolved techniques have been used with great success in X-ray crystallography, especially on ultrafast and fast timescales, and have also seen an increase in adoption for cryoEM as new technologies are being developed (Table 1 ▸).

Over recent years, serial X-ray crystallography has taken the lead in time-resolved structural biology, enabling the characterization of structural changes at high (<2 Å) resolution, providing unprecedented insights into biological mechanisms at the atomic level. Despite these advances, X-ray crystallography can be limiting because the crystal structure can restrict large-scale conformational changes or, alternatively, and terminally, large-scale motions can result in breakup of the crystal before diffraction. CryoEM offers an excellent alternative for studying large conformational changes, especially for big macromolecular complexes. The recent advances in hardware and software in cryoEM have fuelled the interest in dynamic studies, especially for *in situ* studies using cryo-electron tomography (cryoET), combined with cryo-focused ion beam (cryoFIB) milling or lift-out techniques (Zens *et al.*, 2024 ▸; Majumder & Zhang, 2025 ▸). These approaches preserve the native cellular architecture while providing access and characterization to compartments of interest, such as signalling complexes or organelles. CryoET has emerged as a powerful tool for *in situ* studies of macromolecular complexes, bridging the gap between structural biology and cellular biology. While recent studies extended time-resolved approaches to cryoET (Table 1 ▸), there is currently no broadly established workflow for light-activated time-resolved cryoET. Existing implementations are typically tailored to specific biological questions, sample types and the timescales of the conformational changes under investigation.

**Figure 1:**
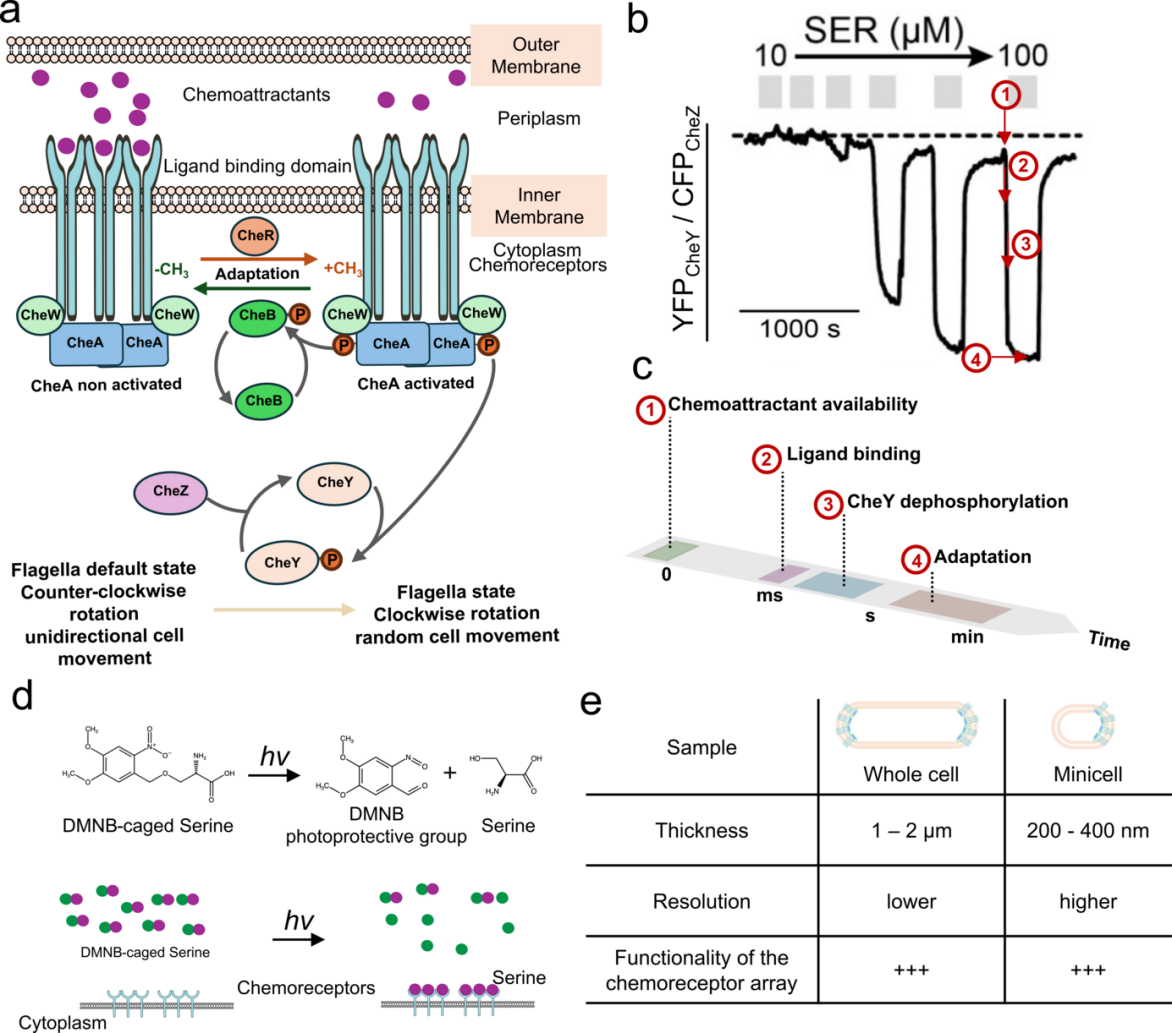
*E. coli* chemotaxis signalling as a model system for time-resolved integrative structural biology. (*a*) Schematic overview of *E. coli* chemotaxis signalling pathway, highlighting the key components and signalling processes. (*b*) FRET-based measurement of pathway activity in *E. coli*. The FRET response, plotted as changes in the YFP_CheY_ to CFP_CheZ_ fluorescence ratio, following sequential additions of serine. Red arrows and numbers indicate key signalling events. (*c*) Summary of chemotaxis signalling events characterized by time-dependent FRET activity shown in (*b*). (*d*) Schematic representation of the mode of action of DMNB-caged serine following light-induced uncaging and its interaction with the chemotaxis system. (*e*) Minicells as an optimal sample system: they retain the chemotaxis functionality while being smaller, enabling cryoET imaging of chemotaxis signalling complexes. Panel (*b*) is adapted from Rowe & Parkinson (2023 ▸) under a CC-BY 4.0 licence.

Here, we describe a light-activation setup and workflow developed for time-resolved cryoET studies of bacterial chemotaxis signal processing. Bacterial chemotaxis is a fundamental process in which motile bacteria sense and respond to chemical gradients in their environment. This behaviour is mediated by a two-component signalling system including a highly ordered two-dimensional (2D) signalling array composed of the chemoreceptors CheA (a histidine kinase) and CheW (a coupling protein), and the effector protein, CheY, which transfers the phosphate group to the flagellar motor and adjusts the motility of the bacterium [Fig. 1 ▸(*a*)]. Trimers of dimers of chemoreceptors bound to a dimer of CheA and two CheWs form the core signalling unit (CSU), the building block of the 2D array. While static structures of the core signalling unit of the array have been elucidated by cryoET, the dynamic nature of the process remains poorly understood (Cassidy *et al.*, 2020 ▸; Hadjidemetriou *et al.*, 2022 ▸). Time-resolved cryoET offers an excellent opportunity to study transient states of signal transduction during bacterial chemotaxis signalling in the cellular context. Such information will provide unprecedented insight into the signal propagation at molecular level and possibly clarify ongoing debates and fill in critical knowledge gaps, such as alternative symmetry of the 2D arrays (Burt *et al.*, 2021 ▸; Muok *et al.*, 2024 ▸) and conformation of the CheA (dipped and undipped conformation) (Cassidy *et al.*, 2020 ▸). There have been single-molecule fluorescence resonance energy transfer (FRET) studies of the dynamics and kinetics of signalling processes upon ligand binding (Paulick & Sourjik, 2018 ▸), which form the basis for selecting the time points for cryotrapping [Fig. 1 ▸(*b*, *c*)]: (1) in the absence of ligand, (2) ligand binding to receptors which occurs on millisecond scale (Jiang *et al.*, 2010 ▸), (3) CheY dephosphorylation happens within few hundreds of milliseconds (Sourjik & Berg, 2002 ▸) and (4) array-level adaptation (methyl­ation/de­methyl­ation) occurs within seconds to minutes (Shimizu *et al.*, 2010 ▸).

Prior kinetic measurements using FRET show that signal propagation through the receptor array and modulation of CheA kinase activity occur on a millisecond timescale. The 150 ms delay used here was therefore selected to capture an early post-ligand binding state, prior to the onset of slower adaptation processes that occur over seconds to minutes. Recently, several time-resolved cryoEM and cryoET approaches have been developed and optimized for purified complexes and in some cases cellular systems, including microfluidic spraying devices, light-coupled plunge freezers and revitrification systems (reviewed in Table 1 ▸). However, *in situ* studies of *E. coli* chemotaxis signalling are not well-suited to microfluidic mixing, which can introduce spatial heterogeneity, or to other approaches requiring complex instrumentation. In contrast, photocaged ligand release enables sub-millisecond and more homogeneous on-grid activation, making it particularly well-suited for probing early signalling events in intact, plunge-frozen bacterial cells while integrating seamlessly with established cryoET sample preparation workflows. This is especially important for cellular samples, where prolonged residence on the grid at room temperature is undesirable.

Driven by the goal of performing time-resolved cryoET of ligand-activated receptor signalling in a near-native environment and on the millisecond timescale, we chose photolysis of a caged ligand as the activation strategy [Fig. 1 ▸(*d*)]. Light-based activation has been widely adopted in structural biology, owing both to advances in light-source technology and to a well-established fundamental understanding of the primary steps of light–matter interactions (Ellis-Davies, 2007 ▸). Moreover, it is an attractive approach for initiating chemical reactions in biological systems because it enables near-instantaneous triggering with minimal perturbation to the sample. The use of photocaged compounds has proven highly effective in probing diverse biological reactions by releasing the molecule of interest, especially in cellular biology (Ellis-Davies, 2007 ▸; Kao, 2006 ▸). This approach requires the introduction of a photocaged compound into an otherwise non-light-sensitive system. Photocaged molecules have also been used to study chemotaxis in *E. coli*, including functional and swarm assays in which chemoeffectors such as serine (Khan *et al.*, 1995 ▸, 1993 ▸, 1992 ▸), glutamate (Brumley *et al.*, 2019 ▸), aspartate (Jasuja *et al.*, 1999 ▸), leucine (Khan *et al.*, 2004 ▸), calcium ions (Tisa & Adler, 1992 ▸), protons (Khan *et al.*, 1995 ▸, 1993 ▸) and oxygen (Yu *et al.*, 2002 ▸) were released. The central principle underlying the use of photocaged molecules is that they are (i) chemically stable and biologically inert prior to photorelease, (ii) uncaged rapidly on sub-millisecond timescales, (iii) loadable into samples and released homogeneously at a defined time point, and (iv) precisely controllable through the properties of the incident light and experimental setup (Ellis-Davies, 2009 ▸; Kao, 2006 ▸).

The reported workflow integrates light activation via photocaged serine to initiate chemotaxis signalling, coupled by rapid plunge-freezing of *E. coli* minicells for time-resolved cryoET studies, using the PORTO laser system (Diamond Light Source) and Vitrobot Mark IV (ThermoFisher). The use of minicells retains the key advantages of studying an intact and functional bacterium, including preservation of native chemoreceptor array architecture and signalling integrity. Importantly, their smaller size compared to bacteria makes minicells more electron-transparent, enabling improved image quality and higher-resolution visualization of the chemo­receptor arrays [Fig. 1 ▸(*e*)]. This approach provides precise temporal control over serine release on the millisecond timescale, enabling the capture of potential intermediate states of the chemotaxis signalling array under near-native conditions. We validate photocage dissociation through photophysical characterization and mass spectrometry, demonstrating robust and reproducible uncaging under defined conditions. In addition, we introduce a novel on-grid and in-line UV–Vis spectroscopy method as a complementary tool to monitor photocage integrity and confirm the photochemical state of the sample prior to vitrification. Together, these developments establish a versatile workflow for studying dynamic biological processes *in situ*, paving the way for a mechanistic understanding of bacterial chemotaxis and other signalling processes.

## 2. Methods

### 2.1. Sample preparation

DMNB-caged serine [*O*-(4,5-di­meth­oxy-2-nitro­benzyl)-l-serine] was purchased from Tocris (Cat. No. 6315). The powder was kept at −20°C until the use of the sample. l-serine was purchased from Sigma-Aldrich (Cat. No. S4500). All chemicals were diluted in 1 × PBS at pH 7.0 (Sigma-Aldrich, Cat. No. P4417-100TAB) or in 10 m*M* KPO_4_ at pH 7.0 (Sigma-Aldrich, Cat Nos. 7778-77-0 and 7758-11-4) and 0.1 m*M* K-EDTA (Sigma-Aldrich, Cat. No. 60-00-4) so that the final concentration is 1 m*M*, and the solutions were kept at 4°C in Eppendorf tubes wrapped with aluminium foil to protect the samples from ambient light.

*E. coli* minicells were obtained from the WM4011 strain (pBAD30-flhDC in WM3433 with ΔclpX::kan fadR::Tn10). To purify minicells, a similar protocol was followed as described by Liu *et al.* (2012 ▸). Briefly, 10 ml of bacterial pre-culture was incubated overnight in Luria–Bertani (LB) broth at 37°C and shaking at 200 r.p.m. Then 1 ml of this pre-culture was transferred to a fresh culture with LB broth in a total volume of 100 ml and cultured at 37°C and shaking at 200 r.p.m. for 4 h to late log phase. The culture was then centrifuged at 10 000*g* for 10 min at 4°C to remove large cells. The pellet was discarded and the supernatant was transferred to a new centrifuge tube and centrifuged at 41 000*g* for 15 min at 4°C to spin down the minicells. The supernatant was discarded and the pellet containing the minicells was suspended in 500 µl of PBS, resulting to an OD_600 nm_ = ∼0.05. The resulting minicells were approximately 427.7 ± 85.1 nm (*n* = 38) and 433.4 ± 96.4 nm (*n* = 106) in diameter, for laser-off and laser-on datasets, respectively, obtained at high purity, and retained chemotaxis signalling function, as previously reported (Burt *et al.*, 2020 ▸).

### 2.2. Photophysical characterization of the DMNB-caged serine

**Figure 2:**
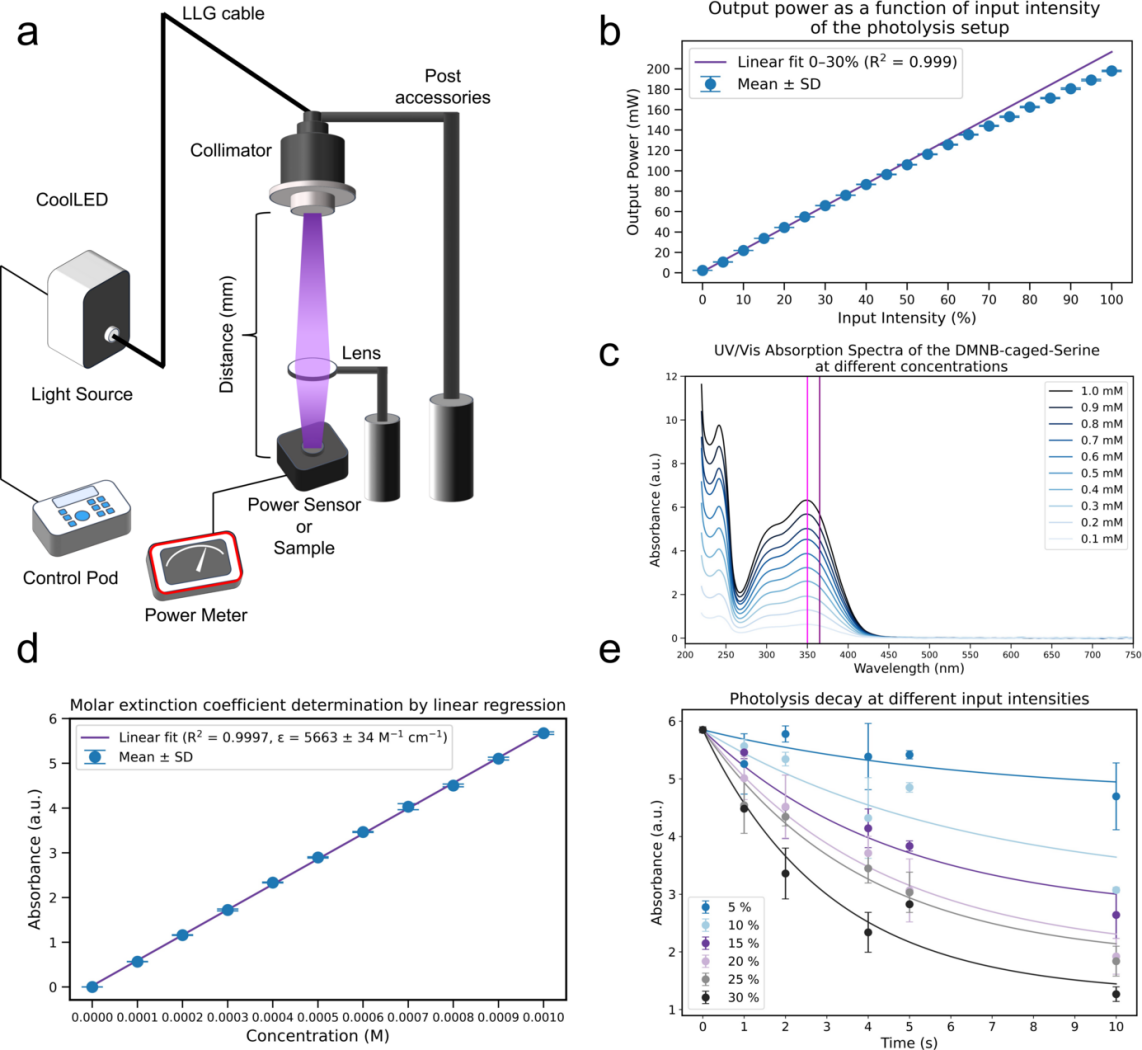
Photophysical characterization of DMNB-caged serine. (*a*) Photolysis setup featuring a 365 nm LED source and collimating/focusing optics. Samples (5 µl) were deposited on paraffin film and positioned at the location normally occupied by the power sensor, as shown in Supplementary Figure S1. (*b*) Measured LED output power (mW) as a function of input intensity (%), demonstrating linear behaviour between 0–30% intensity. (*c*) UV–Vis absorption spectra of the ‘dark’ state of DMNB-caged serine at varying concentrations. Vertical axes represent the maximum wavelength at 350 nm (pink) and the wavelength at which the photophysical characterization was performed (purple). (*d*) Determination of the molar extinction coefficient (ɛ) at 365 nm from the data in (*c*) using linear regression. (*e*) Photolysis kinetics at 365 nm at different LED input intensities (%) within the linear operating regime.

A photolysis setup was designed using the CoolLED pE-4000 illumination system (CoolLED Ltd), an LLG (Liquid Light Guide) fibre (CoolLED pE-1906) and a collimator [LLG5A6 – 5 mm diameter LLG Collimating Adapter for Cerna CSE2100, ARC: 350–650 nm (Thorlabs)]. This combination of LLG and collimator was found to minimize light loss and maximize the light power and fluence delivered to the sample. The wavelength of the CoolLED was set at 365 nm and the input channel intensity varied between 0 and 100%. The output power from the collimator was measured by using a power sensor coupled with a power meter [S405C – Thermal Power Sensor Head, Surface Absorber, 0.19–20 µm, 100 µW – 5 W, 10 mm diameter (Thorlabs)]. The efficiency of the setup was determined as a function of its output power by varying the input intensity at a working distance of 190 mm (from collimator to power sensor). For the final setup [Fig. 2 ▸(*a*)], a plano-convex lens was introduced to the photolysis setup between the collimator and the power sensor to focus the beam in the smallest possible area. The focused beam diameter was 10 mm. Three independent measurements of the output power were taken for each input intensity.

Once the photolysis setup was finalized, 5 µl droplets of 1 m*M* DMNB-caged serine were pipetted on a flat paraffin film on top of the power sensor, in order to ensure that the working distance between the lens and the sample is the same as the distance between the lens and the power sensor used to measure the output power. A photo of the sample droplet on the top of the paraffin film was taken and uploaded to *ImageJ* for further image analysis including drop surface area estimation using the LBADSA *ImageJ* plugin. The droplet properties are displayed in Supplementary Figure S1, showing that the contact angle between the droplet and the paraffin film is about 97.9°. The surface area of the droplet that faces the 365 nm LED light is 3.76 mm^2^ and the respective detection area of the power sensor (5 mm diameter) is 78.5 mm^2^. Thus, the ratio between them is 0.048.

For experimental determination of the rate constant (Section 3.2), illumination times of 0, 1, 2, 4, 5 and 10 s were used. Illumination timing and light-source triggering were controlled using a signal generator, ensuring precise and reproducible exposure times. Each condition was measured in triplicate. The 0% input intensity was a ‘dark’ measurement. For the molar extinction coefficient (ɛ) determination, solutions of decreasing concentration of DMNB-caged serine were prepared ranging from 1 m*M* to 0.1 m*M*. The corresponding UV–Vis spectra were then acquired in triplicate for each concentration. The absorption at 356 nm for each condition was plotted as a function of the concentration, and ɛ_365 nm_ was obtained from the slope of a linear regression fit (Section 3.1).

For the determination of the photochemical quantum yield (*Q*), experiments were performed under conditions of continuous, uniform illumination and low conversion following an approach adapted from Stadler *et al.* (2018 ▸). Here, *Q* is defined as the number of uncaging events per absorbed photon under first-order photolysis conditions. Therefore, *Q* is given by
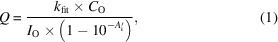
where *k*_fit_ (s^−1^) is the apparent first-order photolysis rate constant obtained from kinetic fitting, *C*_O_ is the initial concentration of DMNB-caged serins (1 m*M*), *I*_O_ is the incident molar photon flux (mol l^−1^ s^−1^) and *A*_i_ is the initial absorbance at 365 nm. The term 
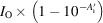
 refers to the fraction of incident photons absorbed by the sample.

The molar photon flux *I*_O_ was calculated from the radiant power delivered to the sample position according to
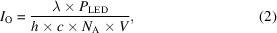
where λ is the CoolLED peak wavelength (365 nm), *P*_LED_ (J s^−1^) is the radiant power on the sample, *h* is Planck’s constant, *c* is the speed of light, *N*_A_ is Avogadro’s constant and *V* is the illuminated reaction volume (5 µl).

Time-dependent UV–Vis absorbance measurements at 365 nm were fitted using a non-linear least-squares fitting of the data to a first-order exponential decay with a non-zero asymptote,
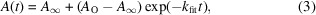
where *A*(*t*) is the absorbance (arbitrary units) at a certain time *t*, *A*_O_ is the initial absorbance (arbitrary units) before the illumination, 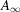 accounts for incomplete conversion within the experimental window, *k*_fit_ (s^−1^) is the rate constant and *t* is the time of illumination (s).

The spectra for the quantum yield and the molar extinction coefficient (ɛ) calculation were recorded with a DS-11 UV–Vis spectrophotometer (DeNovix). All measurements were performed in triplicate. The device was set to the microvolume mode and carried out an automatic baseline correction at 750 nm. For each measurement, 1 µl of the sample was used.

### 2.3. On-grid UV–Vis spectroscopy

To directly validate the photochemical state of the sample immediately prior to vitrification, we developed a novel on-grid UV–Vis spectroscopy approach. This combines the Speed Blot system (Cryo Preparation Limited) and a plunge freezer, with microspectrophotometer hardware developed at Diamond Light Source. The setup is well-suited for characterizing reactions on-grid before plunging the sample in liquid ethane and cryo-trapping the desired intermediate state prior to data collection. Optical spectroscopy, as a complementary method to structural studies, could confirm the state of the system under study, *e.g.* the redox state of a protein after ligand binding or release immediately prior to plunge-freezing.

The modular architecture of the Speed Blot, along with the in-house microspectrophotometer, enables UV–Vis spectroscopy to be performed directly on EM grids. More generally, this setup allows UV–Vis spectroscopy to be used to track reactions initiated either by mixing or light, with the former taking advantage of the mixing capabilities of the Speed Blot itself. This plunge freezer allows the blot, plunge and mix times to be altered in 10 ms steps from 20 ms to seconds. However, for the current light-activation experiments, samples were manually deposited on grids and the CoolLED was used to release serine from the DMNB cage. In the future, we anticipate that the described combination of on-grid spectroscopy and Speed Blot could be used not only for sample preparation for cryoEM but also for cryo-trapping microcrystals for conventional X-ray crystallographic studies.

**Figure 3:**
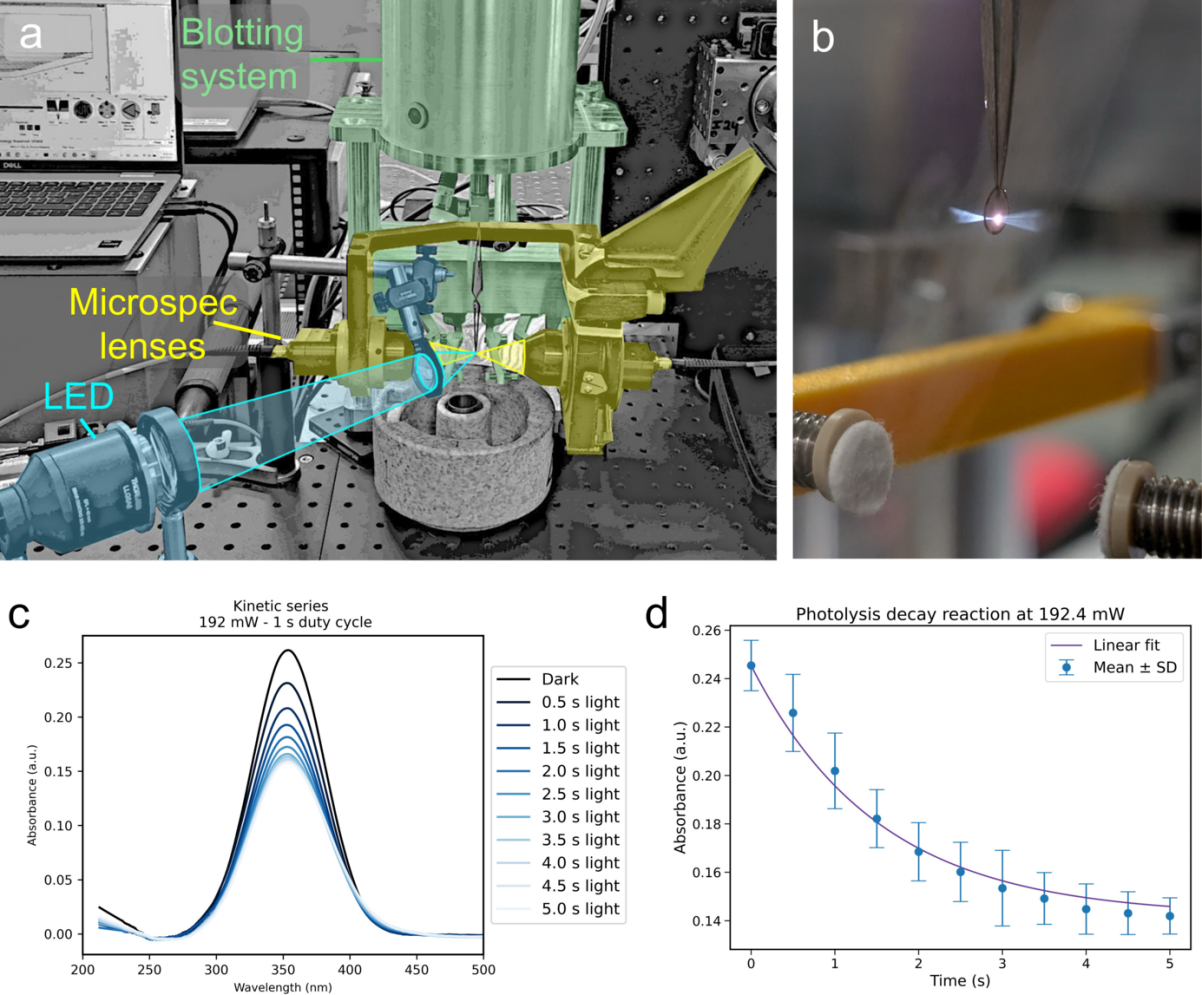
On-grid UV–Vis spectroscopy. (*a*) Overview of the experimental setup, highlighting LED light delivery, the microspectrophotometer and the blotting system in blue, yellow and green, respectively. (*b*) Close-up view of the EM grid positioned above blotters for UV–Vis absorption measurements. (*c*) UV–Vis absorption kinetic series (0.5–5 s total exposure to light) monitoring DMNB-caged serine release at maximum LED power (192.4 ± 0.3 mW, 100% intensity). The pre-illumination (‘dark’) on-grid spectrum of the caged serine is shown in dark blue. (*d*) Photolysis decay kinetics derived from the decrease in absorbance at 365 nm for DMNB-caged serine at maximum LED power.

The integrated setup comprises the microspectro­photometer, the Speed Blot, the CoolLED and a humidifier [Fig. 3 ▸(*a*)]. UV–Vis spectra were measured at room temperature using two reflective objectives over the wavelength range of 250–800 nm. The microspectrophotometer setup uses a Shamrock 303i spectrometer (Andor Technology), a Newton EM CCD detector and a fibre-coupled Xenon light source (Thorlabs). Spectra were an accumulation of 10 exposures each of 10 ms duration. The size of the white light beam was approximately 50 µm in diameter, covering a small area of the grid (Quantifoil R 2/2 on 300 copper mesh) with an ∼3 mm diameter, as shown in Fig. 3 ▸(*b*).

For on-grid photolysis of the DMNB-caged serine, the CoolLED (see Section 2.2) was coupled to the microspectrophotometer, using the same collimator and LLG. However, for these experiments, two lenses were used to focus the 365 nm beam at the sample position on the grid: a collimating and a focusing lens with 190 mm and 35 mm focal length, respectively. The diameter of the UV beam at the sample position was 6 mm, ensuring an overlay with the spectroscopy probe while illuminating the whole grid. The CoolLED was flashed on and off using a signal generator with a duty cycle of 1 s (0.5 s on; 0.5 s off) while UV–Vis spectra were simultaneously recorded continuously in a kinetic series. For each condition, measurements were performed in triplicate, using independently prepared glow-discharged grids to assess reproducibility across grids. For these experiments, 2.5 µl of 1 m*M* sample was pipetted directly on the glow-discharged grids prior to data collection. It was found to be essential to apply a hydro­phobic coating (Rain-X) to the tweezers holding the grids prior to pipetting the sample. Kinetic series (0.5–5 s total exposure) were recorded for the input intensities of 10, 50 and 100%, corresponding to output powers of 21.9 ± 0.5 mW, 104.3 ± 0.6 mW and 192.4 ± 0.3 mW, respectively. Owing to the contribution of the CoolLED light to the observed spectra at 365 nm, data collected in the periods when the CoolLED was on are not shown and were excluded from the analysis. All spectra were processed with an in-house Python script using a Savitzky–Golay filter (polynomial function of degree 1 and window width of 51 data points) for smoothing. The baseline of the smoothed spectra was then corrected using the *IModPoly* method of the *BaselineRemoval* Python library.

### 2.4. Characterizing photo-uncaging of DMNB-caged serine by mass spectrometry

For assessing uncaging, we compared photorelease of DMNB-caged serine with and without laser illumination. Ten times 5 µl of 1 m*M* DMNB-caged serine were irradiated with the PORTO laser using the same parameters described in Section 2.5, and the total volume then diluted in 100 µl PBS. The same concentration and dilution procedure was repeated without laser illumination to provide a second sample set, which also served as a standard along with 1 m*M* serine in PBS. 500 µl of methanol was added to each sample and left overnight in a centrifugal vacuum concentrator. Chemical derivatization was performed to convert metabolites into volatile and thermally stable derivatives for two-dimensional gas chromatography mass spectrometry (GC×GC-MS) analysis, following essentially the protocol as described previously (Yu *et al.*, 2017 ▸) with some modifications. The pellets were re-suspended in 15 µl of 20 mg ml^−1^ meth­oxy­amine–HCl in pyridine. The samples were then incubated and shaken for 90 minutes at 30°C. 10 µl of pyridine and 20 µl of *N*-methyl-*N*-tri­methyl­silyltri­fluoro­acetamide with 1% chloro­tri­methyl­silane were added for each sample and followed by incubation and shaking at 1200 r.p.m. at 60°C for 60 minutes.

Samples were analysed using a Shimadzu GCMS QP2010 Ultra mass spectrometer system coupled to an AOC-20i/s autosampler. Chromatographic separation was achieved by using a dual-column system consisting of an SHM5MS column (30 m × 0.25 mm ID, 0.25 µm film thickness) connected in series to a BPX-50 column (5 m × 0.15 mm ID, 0.15 µm film thickness), providing enhanced separation of derivatized photolysis products. The quantities that were injected for standards were 6 nmol and 0.15 nmol for DMNB-caged serine and serine, respectively. Samples illuminated with or without the laser at 1 µJ were injected at 280°C in splitless mode at 1 µl and spectra recorded at 70 eV (*m*/*z* 45–600, scan speed 5000–20 000 a.m.u.).

Data were processed using *GCMSsolution* software (v2.72/4.20, Shimadzu), *Chromsquare* software (v2.1.6, Shimadzu) and *GC Image* (v2.3) identification employed NIST 11/s, OA_TMS, FA_ME and in-house YUTDI libraries. For peak picking and peak quantitation using the *GCMS­Solution* software (v4.2), we used the following parameters: slope: 2000 min^−1^, width: 0.04 s, min area 100, drift 0 min^−1^ and T. DBL: 1000 min without any smoothing methods.

### 2.5. PORTO laser setup

As an activation trigger for our reactions, we used light from the PORTO instrument (Smyth *et al.*, 2025 ▸), which comprises an industrial-grade femtosecond laser system, an optical parametric amplifier able to deliver laser pulses at wavelengths ranging from 210 nm to 2600 nm and a high-resolution synchronization unit (Light Conversion). Apart from wide wavelength tunability, the system also features adjustable repetition rate, delivering pulses on demand, from a single pulse (300 fs) up to frequencies equal to 53.34 kHz, whilst it can also be externally triggered. The action sequence in the side of the Vitrobot Mark IV (ThermoFisher) and the grid illumination before the plunge-freezing step was initiated by a waveform generator (33521A, Keysight Technologies) which created a TTL (transistor-transistor logic) signal that was sent through the pedal mechanism of the Vitrobot enabling the plunge movement of the arm. The movement of the arm is at the vertical orientation going downwards and is intercepted by an auxiliary setup, the primary role of which was to eliminate the timing uncertainty for the arm plunging, which was found to be of the order of a few tens of milliseconds. The auxiliary setup includes a constant-wave infrared (CW-IR) low-power fibre laser (model S1FC1550, Thorlabs, 1550 nm), a photodiode (DET01CFC/M, Thorlabs), and a set of off-axis parabolic mirrors. The output of the laser is waveguided through an optical fibre and collimated to a size of about 5 mm FWHM. The beam then propagates in free space, via which the arm is going through, prior to being collected via a second off-axis parabolic mirror and focused down to the entrance of a second fibre with its output being sent to a sensitive photodiode (model S1FC1550, Thorlabs). The analogue output of the photodiode is split into two parts. One is fed into an oscilloscope for monitoring, and the other is sent into a delay generator which is connected to a fast camera and to the pulse picker of the main laser amplifier. Upon plunge movement, the collimated IR beam path is interrupted, and the signal drop triggers the formation of a TTL pulse from the delay generator (DG645, Stanford Research System) which triggers the acquisition of the fast camera (Nova S12, Photron) and is sent to the amplifier, releasing the laser light and irradiating the sample. The laser system can deliver a single pulse or a series of pulses, in the form of a burst, at a controllable predefined number and time delay between them, making the experiment extremely reliable and reproducible, providing high temporal accuracy only limited by the capabilities of the Vitrobot and the frame rate of the camera. The beam was 4 mm (FWHM), covering the grid uniformly, and 1 µJ energy per pulse, and for the purposes of the present work a single laser pulse was used. The time points collected were determined by analysing the frames recorded by the camera and computed to be equal to the sum of the illumination time, τ_light_, and the plunge time, τ_plunge_. The timing of laser irradiation and plunge freezing was characterized using a fast camera operating at 12 800 frames per second. The plunge time (τ_plunge_) was 106.7 ± 1.52 ms (*n* = 3) and the laser-to-vitrification delay was directly validated as 150 ± 1.52 ms.

For the EM grid preparation, lacey carbon film on 300 mesh copper grids were used (Agar Scientific). All the grids were subjected to glow discharge using a PELCO easiGlow^TM^ (Agar Scientific) at 30 mA for 30 s. For the laser-on dataset, 9 µl of the suspended minicells in the buffer (see Section 2.1) were mixed homogeneously with 1 µl of 10 m*M* caged serine, resulting to a final concentration of 1 mM of caged serine, thus the photocaged molecule is omnipresent. 3 µl of this new mixture (minicells and 1 m*M* of caged serine) were loaded on the grid and then mounted on the Vitrobot. Grids were blotted manually before the light irradiation with the PORTO laser system whilst the entire area around the grid and inside the Vitrobot was quasi-saturated with humidity. As the laser beam covers the whole grid, serine release occurs uniformly across the area, and thus triggering of chemotaxis signalling is synchronized. For the laser-off (control) dataset, 3 µl of minicells in PBS were loaded on the grid, blotted and plunge-frozen without any laser illumination.

Laser-induced heating of the EM grid upon laser irradiation was estimated using a heat-capacity model along with the experimentally validated copper EM grid parameters for cryoEM applications (Bhattacharjee *et al.*, 2023 ▸). The heat-capacity model gives the laser-induced temperature increase as
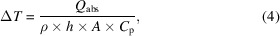
where *Q*_abs_ is the absorbed optical energy, ρ the copper density, *h* the grid thickness, *A* the illuminated area of the grid and *C*_p_ the specific heat capacity of the copper grid. The following values were used, all taken directly from Bhattacharjee *et al.*, as they are specific to the copper EM grid: ρ = 8960 kg m^−3^, *h* = 25 µm, *A* = 7.07 × 10^−6^ m^2^, and *C*_p_ = 377 J kg^−1^ K^−1^. The absorbed optical energy (*Q*_abs_) is the product of the copper absorptivity (α) and the incident energy (*E*_inc_). An absorptivity (α) of 0.6 was used based on the experimental validation reported by Bhattacharjee *et al.* Under our experimental conditions [single 300 fs pulse with 1 µJ incident energy (*E*_inc_) at 350 nm], the absorbed optical energy (*Q*_abs_) is 0.6 µJ, yielding an estimated temperature rise of ∼1 × 10^−3^ K per pulse, indicating minimal laser-induced heating under the single-pulse low fluence conditions used in this study.

### 2.6. cryoET data collection and image processing

Tilt series were collected on a Titan Krios I (eBIC, Diamond Light Source) cryo transmission electron microscope operated at 300 kV and equipped with a K3 detector (Gatan) operating in correlated-double sampling (CDS) mode and a slit of 20 eV. Tilt series were recorded at a nominal magnification of 64 000×, which corresponds to a calibrated physical pixel size of 1.67 and 1.63 Å, for laser-off and laser-on datasets, respectively, using the dose-symmetric scheme with tilt span −57° to 57° and 3° increments. The nominal defocus range was 2.5 to 5.5 µm, and the total dose was 170 e^−^ Å^−2^. Each micrograph was fractionated into ten movie frames. In total, 38 out of 42 and 106 out of 174 tilt series that were collected, for laser-off (control) and laser-on datasets, respectively, were selected for downstream image processing. Movie frames were motion-corrected using *MotionCor2* (Zheng *et al.*, 2017 ▸), followed by CTF estimation within individual tilts using *CTFFIND4* (Rohou & Grigorieff, 2015 ▸). Both preprocessing steps were performed by the on-the-fly tomography processing pipeline *PATo* (Horstmann *et al.*, 2024 ▸) at eBIC. Tilt-series alignment and reconstruction were performed using *AreTomo2* (Zheng *et al.*, 2022 ▸). Template matching was performed using *emClarity* (Himes & Zhang, 2018 ▸) to select chemotaxis C3-centred core signalling units. The template used in this step was the cryoET-derived map EMD-10160 (Burt *et al.*, 2020 ▸). A total of 17 819 and 40 525 particles for laser-off and laser-on datasets, respectively, were extracted and averaged with *emClarity* at bin level 5. No further classification was performed.

## 3. Results

### 3.1. *Ex situ* photophysical characterization of the DMNB-caged serine

Photophysical characterization of DMNB-caged serine includes determination of the molar extinction coefficient, kinetics and quantum yield of the kinetics of the photorelease. Towards this, we designed a photolysis setup featuring a 365 nm LED source and collimating/focusing optics [Fig. 2 ▸(*a*), Section 2.1]. The efficiency of the LED output power (mW) was measured as a function of input intensity (%) [Fig. 2 ▸(*b*)]. Linearity between input values and output powers is observed between 0 and 30%, beyond that the relationship proved to be nonlinear [Fig. 2 ▸(*b*), Supplementary Table S1]. For the downstream *ex situ* photophysical characterization, we therefore used only this range of LED input intensities, where the setup is calibrated and operates linearly *i.e.* the input intensity is proportional to the output power. The UV–Vis absorption spectra of the ‘dark’ state (*i.e.* without any illumination) of DMNB-caged serine displayed a maximum absorbance wavelength (λ_max_) at 350 nm [Fig. 2 ▸(*c*)], characteristic of the *o*-nitro­benzyl moiety with a shoulder near 300 nm corresponding to π–π* transitions of the meth­oxy substituents (Görner, 2005 ▸; Weinstain *et al.*, 2020 ▸). Because the available LED source was emitted at 365 nm, and not at a shorter wavelength, absorbance measurements and photolysis were performed at this wavelength. To estimate the molecular extinction coefficient (ɛ) at 365 nm, spectra at different concentrations from 0.1 to 1 m*M* of DMNB-caged serine were recorded [Fig. 2 ▸(*c*)]. According to the Beer–Lambert law, the absorbance (*A*) is proportional to the concentration (*C*) if the path length (*l*; 1 cm here) and the molar extinction coefficient are constant (*i.e*. *A* = ɛ × *l* × *C*). By plotting absorbance at 365 nm *versus* concentration [Fig. 2 ▸(*d*)], a molar extinction coefficient of ɛ_365 nm_ = 5663 ± 34 *M*^−1^ cm^−1^ was obtained. The extinction coefficient at the maximum absorbance wavelength 350 nm was also measured as ɛ_350 nm_ = 6309 ± 42 *M*^−1^ cm^−1^.

For the quantum yield (*Q*) determination, we follow the kinetics of the photorelease in accordance with the protocol developed by Stadler *et al.* (2018 ▸), see Section 2.2. The photolysis kinetics and rate constant (*k*_fit_) were monitored by time-resolved UV–Vis spectroscopy at 365 nm using the LED-based photolysis setup described in Section 2.2 [Fig. 2 ▸(*a*, *e*), Table S2]. Under the linear operating conditions (5–30% of the input intensity) of the CoolLED system, the rate constant (*k*_fit_) increases linearly with incident photon flux. The quantum yield *Q*, obtained from the slope of this linear fit (Supplementary Figure S2, Supplementary Table 3), was 0.102 ± 0.015. Therefore, the photophysical characterization of the photocage serine using this setup allowed determination of the molar extinction coefficient and quantum yield values, which are consistent with the previously reported values (Monteiro *et al.*, 2021 ▸).

### 3.2. On-grid UV–Vis spectroscopy as an integrated validation tool for time-resolved studies

We extended the photophysical characterization directly to the sample on the EM grid, giving rise to a new on-grid UV–Vis spectroscopy approach [Fig. 3 ▸(*a*, *b*), Supplementary Video 1]. This approach enables closer investigation of the photocage release under cryoET-relevant sample preparation conditions. UV–Vis absorption spectra were collected on grids to characterize the release of the DMNB-caged serine with 365 nm light. Fig. 3 ▸(*c*) shows the gradual decrease in the maximum peak of the caged serine at 365 nm for the kinetic series (0.5–5 s LED exposure) recorded at the maximum output power (192.4 ± 0.3 mW). The UV–Vis spectra of the DMNB-caged serine recorded on-grid showed a maximum absorbance at 350 nm, consistent with spectra acquired using the commercial DS-11 spectrophotometer [Fig. 2 ▸(*c*)], indicating similar photophysical behaviour under both measurement conditions. The primary difference between the two spectrophotometers is that below ∼300 nm, little signal could be recorded with the on-grid microspectrophotometer. This limitation of the latter arises from the reduced output of the Xe lamp at wavelengths less than 300 nm, combined with optical losses in the fibres and lenses, rather than from changes in the sample itself. Additionally, the spectra [Fig. 3 ▸(*c*)] showed that the EM grid structure allows sufficient transmission of light for on-grid sample characterization, and the grid itself does not contribute significant spectral features. The ‘dark’, pre-illumination spectrum of the DMNB-caged serine is shown in black, followed by a gradual decrease of the 365 nm absorbance peak after each 0.5 s exposure to the 365 nm LED light, indicating successful uncaging on the grid [Fig. 3 ▸(*c*)].

The corresponding photolysis decay was fitted using a first-order exponential decay with a non-zero asymptote [Fig. 3 ▸(*d*), equation (3)], yielding an apparent rate constant (*k*_fit_) of 0.65 ± 0.03 s^−1^ (decay half-time = 1.06 s). Photo-uncaging is again photo-power-dependent as shown for both the *ex situ* [Fig. 2 ▸(*e*)] and on-grid setups (Fig. 3 ▸, Supplementary Figure S3), with rate constants in both approaches being of the order of seconds. This slow photolysis (seconds in scale), measured by both *ex situ* and on-grid UV–Vis spectroscopy with an LED light under continuous and low-intensity illumination, is likely limited by the photon flux (or output power) rather than the intrinsic photochemical uncaging event, which occurs on femtosecond-to-microsecond timescales following photon absorption.

### 3.3. Assessing femtosecond-pulsed laser induced photorelease with mass spectrometry

To overcome the limited photorelease efficiency achieved with the CoolLED system and to increase photon flux, we employed the femtosecond-pulsed PORTO laser available at Diamond Light Source as the photoactivation source for rapid releasing of DMNB-caged serine in our time-resolved cryoET experiment.

**Figure 4:**
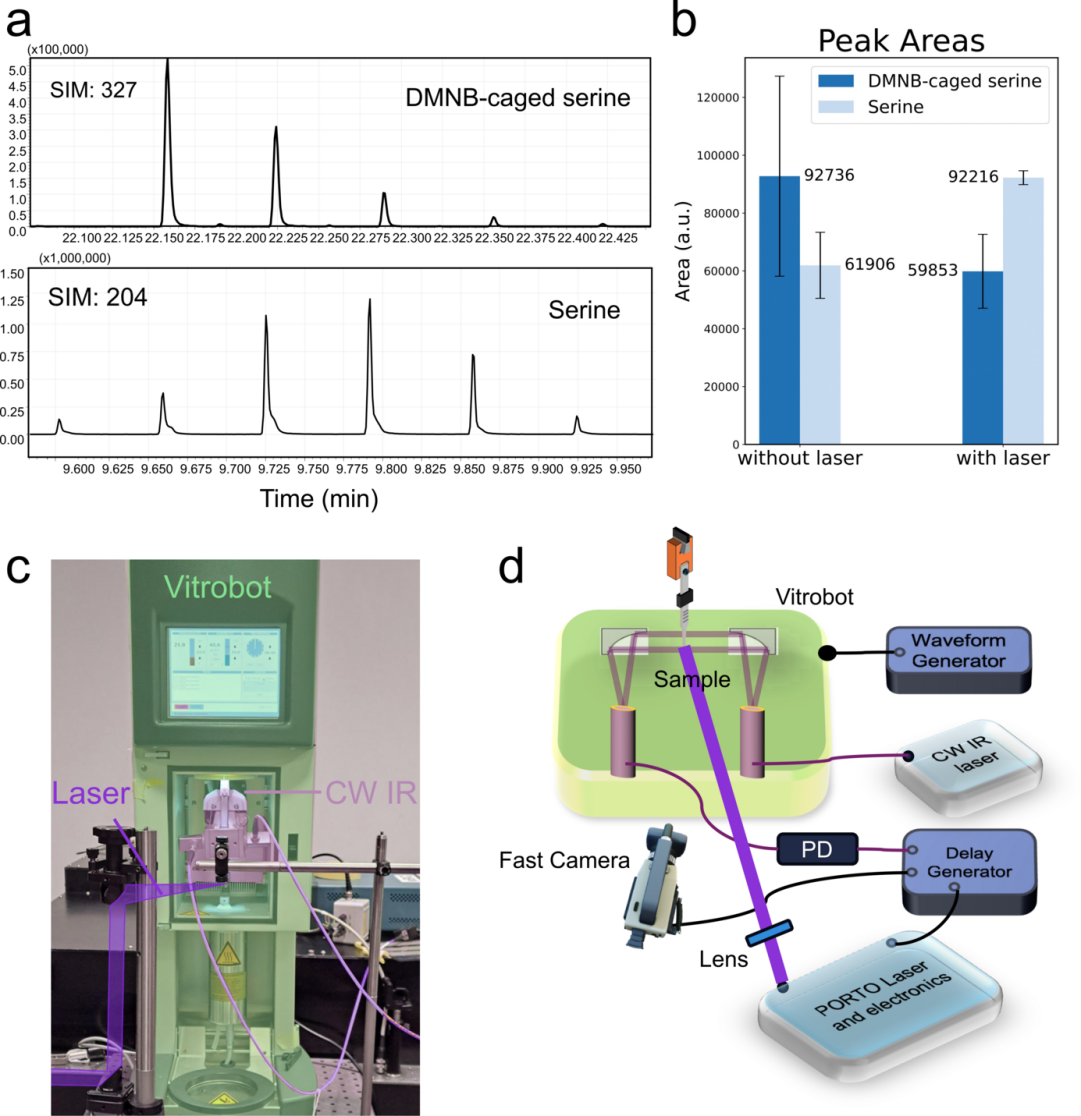
Photolysis setup with the PORTO laser and Vitrobot and GC×GC-MS analysis. (*a*) GC×GC-MS single ion monitoring (SIM) chromatograms showing retention times for DMNB-caged serine and free serine following PORTO laser illumination at 1 µJ of energy. (*b*) The panel shows the integration of the peak areas for DMNB-caged serine (*m*/*z* = 327) and free serine (*m*/*z* = 204) before and after laser illumination. The ratios of the integrated peak areas (serine/DMNB-caged serine) are ∼0.7 and ∼1.6 for the samples without and with laser illumination, respectively. (*c*) Overview of the experimental setup with a Vitrobot, highlighting key components and the laser beam path. (*d*) Schematic of the experimental apparatus, showing the PORTO laser used for sample irradiation, a fast camera providing high temporal resolution, and the optical assembly comprising a continuous-wave (CW) IR laser, optical fibres, a pair of off-axis parabolic mirrors and a photodiode (PD). The photodiode provides the trigger signal to initiate the measurement sequence, while the Vitrobot controller initiates the motion sequence; associated electronic control units are also indicated.

Firstly, we analysed separately the standards, DMNB-caged serine and serine only, using two-dimensional gas chromatography mass spectrometry (GC×GC-MS) and characterized the retention time as 22 s (*m*/*z* = 327) and 9 s (*m*/*z* = 204), respectively [Fig. 4 ▸(*a*), Supplementary Figure S4]. We next examined the effect of photolysis on the release of serine using the PORTO laser system by comparing the chromatographic peak areas obtained without illumination (negative control) and following 1 µJ laser illumination. The integrated peak area ratio of serine to DMNB-caged serine in the non-illuminated sample is ∼0.7 ± 0.1 [Fig. 4 ▸(*b*)]. Upon illumination, the DMNB-caged serine peak is substantially diminished, while the serine peak increases, yielding an area ratio of ∼1.6 ± 0.3 [Fig. 4 ▸(*b*)]. This increase of the ratio was statistically significant (Welch’s *t*-test, *p* = 0.030) and reflects efficient serine uncaging under the experimental illumination condition.

Starting from an initial concentration of 1 m*M* DMNB-caged serine, the serine/DMNB-caged serine ratio increases in the laser illumination condition (from 0.7 to 1.6), which corresponds to the release of free serine well above the 100 µ*M* required to elicit a chemotactic response [Fig. 1 ▸(*b*)], as well as above the reported half-maximal serine response of the Tsr receptor (*K*_1/2_ = 19 µ*M*) (Mowery *et al.*, 2015 ▸). This establishes suitable conditions for downstream time-resolved cryoET of *E. coli* chemotaxis signalling. A more extensive energy flux titration may be informative in future experiments, particularly when designing light-activation workflows for cryoEM, to ensure excitation conditions remain within the linear regime.

### 3.4. Towards time-resolved cryoET of *E. coli* minicells

Since uncaging of serine by the PORTO laser at 1 µJ is sufficient, we built a photolysis setup including the PORTO laser system coupled with the Vitrobot. We synchronized the illumination and plunging to achieve a nominal delay of ∼150 ms between the photocage release and the plunge freezing [Fig. 4 ▸(*c*, *d*); Section 2.5]. In practice, the time resolution of this setup is constrained by the Vitrobot mechanics, which is the plunging time that was characterized by the fast camera at ∼100 ms. The setup provides a robust reproducible time-resolved cryoEM sample preparation using a pulsed laser with grid-wide coverage. Serine uncaging with specific laser parameters was measured on-grid using GC×GC-MS (Section 3.3). The time point of ∼150 ± 1.52 ms upon serine binding places the experiment within the early signalling regime of chemotaxis when serine-binding-induced downstream signalling is initiated [Fig. 1 ▸(*b*)].

**Figure 5:**
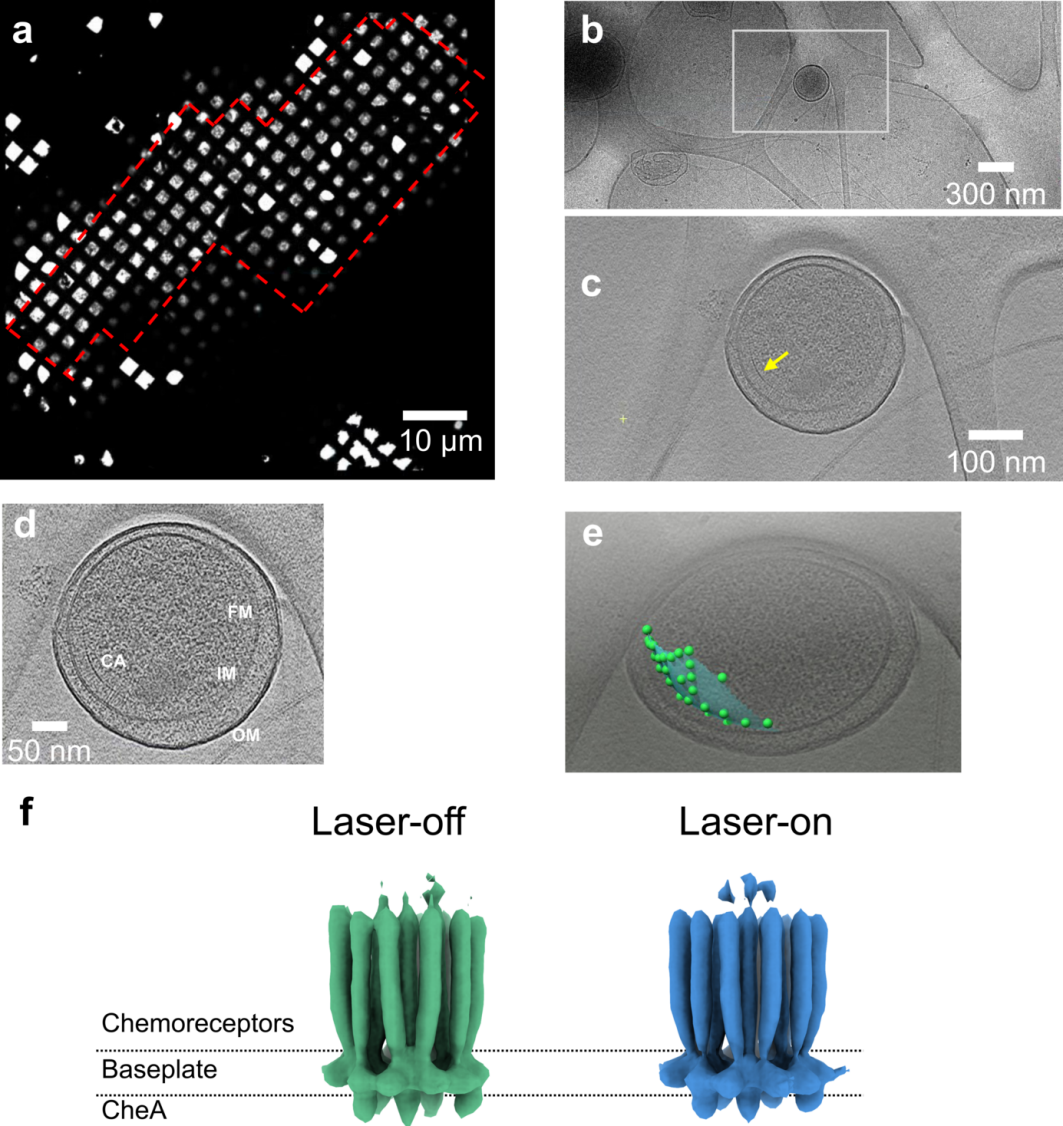
Time-resolved chemotaxis signalling sample preparation using *E. coli* minicells. (*a*) Atlas view (145× magnification) of a representative EM lacey carbon grid prepared using the photolysis setup integrating the PORTO laser and Vitrobot. The sample loaded on the grid consists of purified minicells and DMNB-photocaged serine. The grid was plunge-frozen with a delay of ∼150 ms following light irradiation. Grid squares within the highlighted region (red dashed outline) were selected for cryoET data collection. (*b*) Representative grid overview (8700× magnification) used to assess minicell distribution and ice thickness. (*c*) Tomographic slice from the region highlighted by the dashed rectangle in (*b*). The yellow arrow points to a chemotaxis array. (*d*) Enlarged view of a minicell from (*c*), showing identifiable structural features including the outer membrane (OM), inner membrane (IM), chemotaxis array (CA) and flagellar motor (FM). (*e*) Template-matching results (green spheres) obtained using *emClarity*, showing matched hexagonal patterns of the chemotaxis array overlaid on the tomogram region corresponding to (*d*). (*f*) Preliminary subtomogram averaged maps of the tri-CSU complex at ∼20 Å resolution for the laser-off and laser-on datasets.

A representative atlas of the laser-on data [Fig. 5 ▸(*a*)] shows uniform ice coverage in the middle area of the grid, while a higher magnification overview [Fig. 5 ▸(*b*)] confirms appropriate ice thickness and minicell integrity and distribution. It is evident that the pulsed laser beam did not obviously damage either the grid’s support film or the sample. CryoEM micrographs showed predominantly intact minicells with a diameter of 433.4 ± 96.4 nm (*n* = 106). Tomographic sections reveal bacterial architecture components consistent with healthy minicells [Fig. 5 ▸(*c*) and 5(*d*)]. Specifically, visible chemotaxis arrays appear along the inner membrane. Template matching using a cryoET-derived map (EMD-10160) identifies C3-centred trimers of the CSU which represent the basic building blocks of the chemotaxis array. These units form an extended hexagonal array as expected [Fig. 5 ▸(*e*)]. A preliminary subtomogram averaged (STA) map at 20 Å resolution [Fig. 5 ▸(*f*)] shows features similar to previously reported STA structures (Burt *et al.*, 2020 ▸). We carried out a parallel control experiment with the PORTO laser off, and the preliminary STA map from the laser-off dataset at 20 Å resolution appears very similar to that from the laser-on dataset [Fig. 5 ▸(*f*)]. In both maps, the same features are visible: chemoreceptors, the baseplate (consisting of CheA and CheW), and the CheA. At this resolution, no structural difference can be resolved between the two maps and they are qualitatively comparable to previously reported architecture from *E. coli* minicell studies (Burt *et al.*, 2020 ▸). Further extensive data collection and detailed classification will be required to analyse the conformations of the CSU with and without PORTO laser excitation. Nevertheless, the consistency of STA maps with and without laser irradiation at ∼20 Å resolution suggests that the workflow preserves sample and structure integrity and provides a foundation for classifying transient states in future studies.

## 4. Discussion and perspectives

In this study, we establish an integrated workflow for light-triggered, millisecond time-resolved *in situ* cryoET in intact bacterial cells. Using DMNB-caged serine and *E. coli* minicells, we demonstrate reproducible and efficient photorelease of serine uncaging across multiple independent validation methods, including *ex situ* photophysical characterization, on-grid UV–Vis spectroscopy and GC×GC-MS. Coupling the femtosecond PORTO laser system to the Vitrobot plunge-freezing device enables grid-wide, synchronous triggering with a directly measured laser-irradiation-to-vitrification delay of ∼150 ms. This time window is placed at the fast side of the chemotaxis signalling dynamics, representing a significant and promising advance over static cryoET approaches, as it enables triggering under near-native conditions in the cell membrane without chemical inhibitors or mutations that could add bias in structural states. Importantly, the illumination regime (a single 300 fs pulse of 1 µJ) introduces negligible heating and preserves cellular integrity, resulting in vitrified samples suitable for *in situ* cryoET analysis.

At the resolution accessible in the present study (∼20 Å resolution), no laser-induced structural damage effects were observed. STA revealed intact minicells and well-reserved chemosensory array architecture in the laser-on dataset, comparable to the laser-off control. Template matching and preliminary STA recover core signalling units arranged in extended hexagonal arrays, consistent with previously reported static structures. These results validate the technical feasibility of combining photocaged ligand activation with time-resolved cryoET in intact cells and demonstrate that the data quality is sufficient for downstream subtomogram-based analyses.

Although photorelease of serine was independently confirmed by UV–Vis spectroscopy and GC×GC-MS, no biologically relevant structural differences between the laser-on and laser-off datasets were detected at the current resolution. Several factors likely limit interpretability at this stage. First, the STA maps are derived from relatively small datasets. Second, any ligand-induced conformational changes may occur below the spatial resolution achieved here. Third, early signalling events are likely associated with increased conformational heterogeneity within the chemosensory array. The coexistence of multiple signalling states can reduce the effectiveness of STA and necessitates further classification to resolve distinct conformational classes (Briggs, 2013 ▸). Resolving distinct signalling intermediates and addressing mechanistic questions will require more extensive datasets and comparative analyses across multiple time points and experimental controls in future studies.

Beyond bacterial chemotaxis, the present workflow helps define a blueprint for dynamic structural studies in cells and provides several avenues for further applications, implementations and developments in diverse biological systems. The step-by-step characterization of a photocaged molecule can provide a basis and encouragement to the community to expand biochemical triggers, while the same strategy can be extended to other small organic molecules, nucleotides (*e.g.* ATP), inhibitors or second messengers. The temporal resolution of the current setup is restricted by the mechanical plunging of the Vitrobot. Integrating the photocage strategy with faster freezing devices like cryojets, Spotiton/Chameleon or Speed Blot could push accessible timescales from a few seconds to 20 ms, and as fast as a few microseconds by using fast devitrification and revitrification approaches (Harder *et al.*, 2023 ▸; Straub *et al.*, 2025 ▸).

Lastly, we report a new on-grid UV–Vis spectroscopy approach which can be used in-line with a high-speed blotting system. This setup expands the capabilities of time-resolved studies by introducing a potential fourth dimension of analysis. This provides an orthogonal, real-time validation of the triggering event and strengthens the interpretability of the structural data. Beyond photocaged ligands, this approach is broadly applicable to other spectroscopically tractable systems, including redox-active proteins, metalloproteins and light-capturing proteins. Such cross-modal verification increases confidence in time-resolved experiments and provides strong support for the interpretation of observed structural states.

## Supplementary Material

Supplementary figures and tables, and full caption for video. DOI: 10.1107/S2052252526005324/hen5002sup1.pdf

Supplementary Video 1: On-grid UV-Vis spectroscopy coupled with a Speed Blot in action. DOI: 10.1107/S2052252526005324/hen5002sup2.mp4

## Figures and Tables

**Table 1:** Time-resolved cryoEM vitrification methods sorted by date

Year	System	Time resolution	Reference
1989	Manual mixing	3–6 s	Siegel *et al.* (1989 ▸)
1990	Electric pulse delivery	3 ms – 10 s	Chang & Reese (1990 ▸)
1993	Light pulse delivery	10–20 ms	Subramaniam *et al.* (1993 ▸)
1994	On-grid mixing	1–100 ms	Berriman & Unwin (1994 ▸)
1995	On-grid mixing	10 ms	Walker *et al.* (1995 ▸)
2009	On-grid mixing	7–70 ms	Adams *et al.* (2009 ▸)
2009	Microfluidic mixing and spraying	9.4 ms	Barnard *et al.* (2009 ▸)
2012	On-grid mixing	10 ms	Unwin & Fujiyoshi (2012 ▸)
2014	Microfluidic mixing and spraying	9.4–34 ms	Shaikh *et al.* (2014 ▸)
2015	Microfluidic mixing and spraying	60–140 ms	Chen *et al.* (2015 ▸)
2019	Microfluidic mixing and spraying	20–600 ms	Kaledhonkar *et al.* (2019 ▸)
2019	Microfluidic mixing and spraying	24–60 ms	Fu *et al.* (2019 ▸)
2019	Microfluidic mixing and spraying	15 ms	Kontziampasis *et al.* (2019 ▸)
2020	Light pulse delivery to release caged compound	70 ms	Yoder *et al.* (2020 ▸)
2020	On-grid mixing	90–150 ms	Dandey *et al.* (2020 ▸)
2020	Microfluidic mixing and spraying	30–1360 ms	Mäeots *et al.* (2020 ▸)
2021	Microfluidic mixing and spraying	7–640 ms	Klebl *et al.* (2021 ▸)
2023	Microfluidic mixing with on-demand jetting	10 ms	Torino *et al.* (2023 ▸)
2023	Rapid revitrification coupled with cage release	30 µs	Harder *et al.* (2023 ▸)
2023	Manual mixing	0.5–15 min	Bodrug *et al.* (2023 ▸)
2024	Manual mixing	5–17 s	Papasergi-Scott *et al.* (2024 ▸)
2024	Mix-and-inject jet stream	264–1315 ms	Yoniles *et al.* (2024 ▸)
2025	Light activation	100 and 4.6–4.8 ms	Kroll *et al.* (2025 ▸)
