# Supplementary material for: Towards light-coupled sample preparation for time-resolved cryoEM studies
Source: IUCrJ. 2026 Jun 29;13(Pt 4):395–408. doi: 10.1107/S2052252526005324 (PMC13324601; doi:10.1107/S2052252526005324)
Supplement: Supplementary file 1 [file m-13-00395-sup1.pdf]

IUCrJ

Volume 13 (2026)

Supporting information for article:

Towards light-coupled sample preparation for time-resolved cryoEM studies

Kyprianos Hadjidemetriou, Sofia Jaho, Pierre Aller, Zhanru Yu, Benedikt M. Kessler, Stephen P. Muench, Nikil Kapur, Gabriel Karras, Robin L. Owen and Peijun Zhang

Table S1 Output power (mW) of the photolysis setup (Section 2.2) measured at the sample position for various input intensities (%).

Input intensity (%)	Output power (mW)				
	Measurement 1	Measurement 2	Measurement 3	Mean	Standard deviation
0	2.4	2.3	2.3	2.3	0.05
5	10.7	10.5	10.5	10.6	0.09
10	21.9	21.9	21.9	21.9	0.00
15	33.7	33.7	33.7	33.7	0.00
20	44.4	44.5	44.4	44.4	0.05
25	54.9	54.9	54.8	54.9	0.05
30	65.7	66.1	65.9	65.9	0.16
35	75.8	76.2	76.1	76.0	0.17
40	86.4	86.9	86.7	86.7	0.21
45	96.0	96.7	96.5	96.4	0.29
50	105.6	106.3	106.2	106.0	0.31
55	115.7	116.6	116.4	116.2	0.39
60	125.1	126.0	125.8	125.6	0.39
65	134.8	135.9	135.7	135.5	0.48
70	143.3	144.6	144.3	144.1	0.56
75	152.2	153.6	153.3	153.0	0.60
80	161.6	163.2	162.8	162.5	0.68
85	170.4	171.9	171.6	171.3	0.65
90	179.5	181.2	180.9	180.5	0.74
95	187.9	189.8	189.4	189.0	0.82
100	197.1	198.6	198.3	198.0	0.65

Table S2 Fitting parameters of the photolysis decay kinetics analysis (Section 3.1) and the fitting equations at each output power of the light source on the sample.

**Input intensity (%)**	5	10	15	20	25	30
**Half-time (s)**	4.532	4.375	3.174	2.987	2.677	2.134
**Time constant (s)**	6.538	6.312	4.579	4.310	3.862	3.079
**Rate constant (s^{-1})**	0.153	0.158	0.218	0.232	0.259	0.325
**R^2**	0.608	0.861	0.960	0.977	0.969	0.955
**Std. Dev. of rate constant (s^{-1})**	0.053	0.034	0.024	0.019	0.024	0.038

**Input intensity (%)**	**Fitting equations**
5	$A(t) = 4.697 + (5.850 - 4.697) \cdot \exp(-0.153 \cdot t)$
10	$A(t) = 3.073 + (5.850 - 3.073) \cdot \exp(-0.158 \cdot t)$
15	$A(t) = 2.640 + (5.850 - 2.640) \cdot \exp(-0.218 \cdot t)$
20	$A(t) = 1.921 + (5.850 - 1.921) \cdot \exp(-0.232 \cdot t)$
25	$A(t) = 1.839 + (5.850 - 1.839) \cdot \exp(-0.259 \cdot t)$
30	$A(t) = 1.266 + (5.850 - 1.266) \cdot \exp(-0.325 \cdot t)$

Table S3 Parameters used for the calculations in Equations 2 and 3.

	**Input intensity of CoolLED (%)**					
	5	10	15	20	25	30
P_{LED} (on the droplet) (J s^{-1})	3.650E-07	3.650E-07	3.650E-07	3.650E-07	3.650E-07	3.650E-07
I_O (mol L^{-1} s^{-1})	5.077E-04	1.049E-03	1.614E-03	2.127E-03	2.630E-03	3.156E-03
k_{fit} (s^{-1})	0.153	0.158	0.218	0.232	0.259	0.325
C_O (mol L^{-1})	1.000E-03	1.000E-03	1.000E-03	1.000E-03	1.000E-03	1.000E-03
k_{fit}*Co (mol.L^{-1}.s^{-1})	1.530E-04	1.580E-04	2.180E-04	2.320E-04	2.590E-04	3.250E-04
A_i (a.u.)	5.850E+00	5.850E+00	5.850E+00	5.850E+00	5.850E+00	5.850E+00
$(1-10^{-Ai})$	1.000E+00	1.000E+00	1.000E+00	1.000E+00	1.000E+00	1.000E+00
$Io(1-10^{-Ai})$	3.098E-04	6.400E-04	9.848E-04	1.297E-03	1.604E-03	1.926E-03

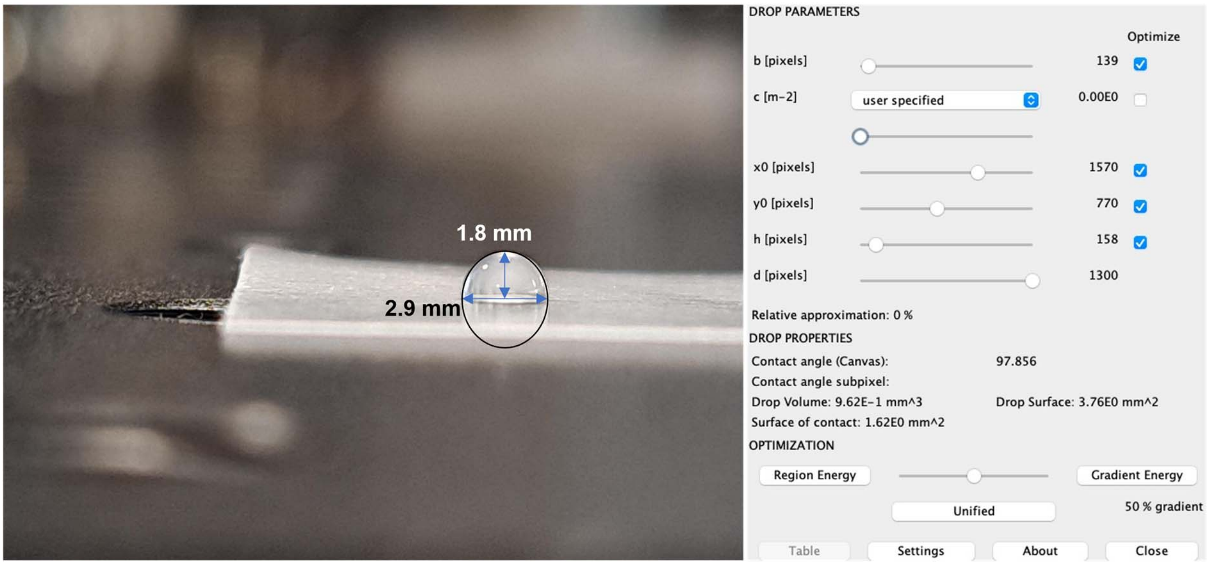

Figure S1 ImageJ droplet analysis. The left panel shows the selected area of the droplet to be analysed in green. On the right panel, the parameters of the analysis are indicated.

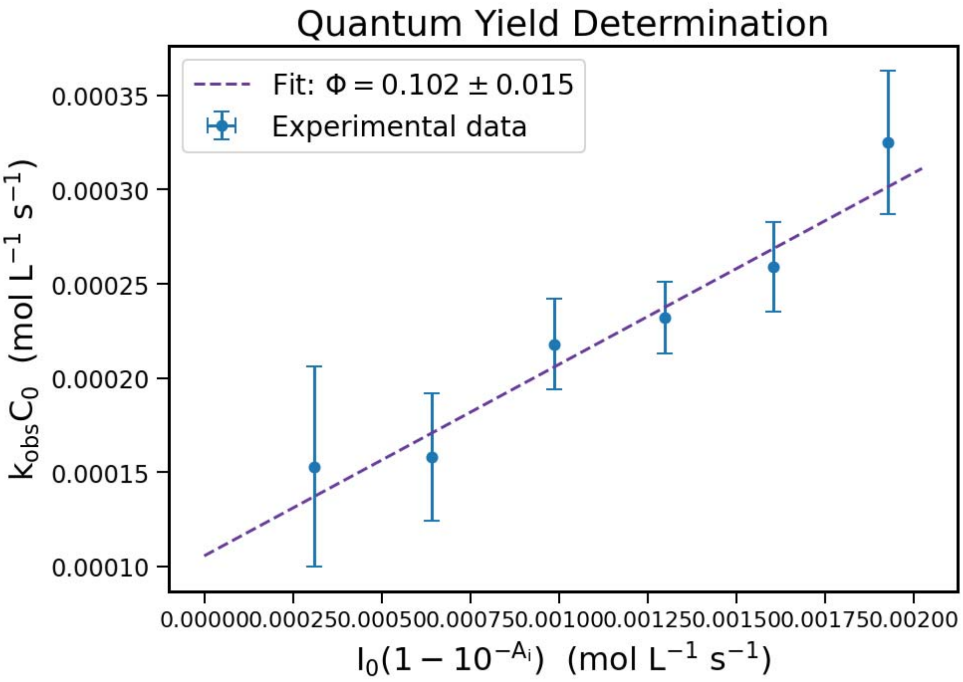

Figure S2 Quantum yield determination of DMNB-caged serine at 365 nm. Plot of the parameters k_{fit} x Co as a function of the initial absorbance at 365 nm. The slope represents the quantum yield (Φ). The linear regression fit of the data displays a standard deviation error of +/- 0.015. The data of this plot were calculated using Equations 2 and 3.

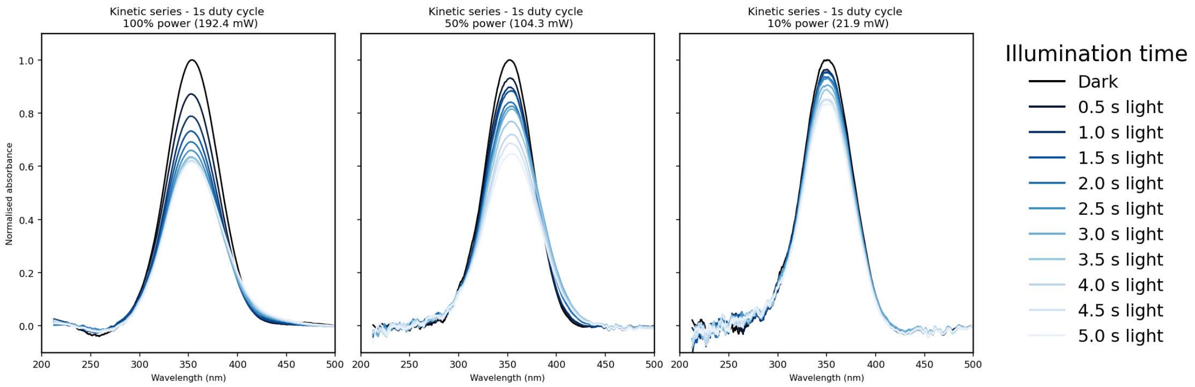

Figure S3 UV-Vis absorption spectra of the DMNB-caged serine collected on-grid as a kinetic series (0.5 - 5 s) of 365 nm LED illumination at a) 100% intensity (192.4 ± 0.3mW), b) 50% intensity (104.3 ± 0.6 mW), and c) 10% intensity (21.9 ± 0.5mW).

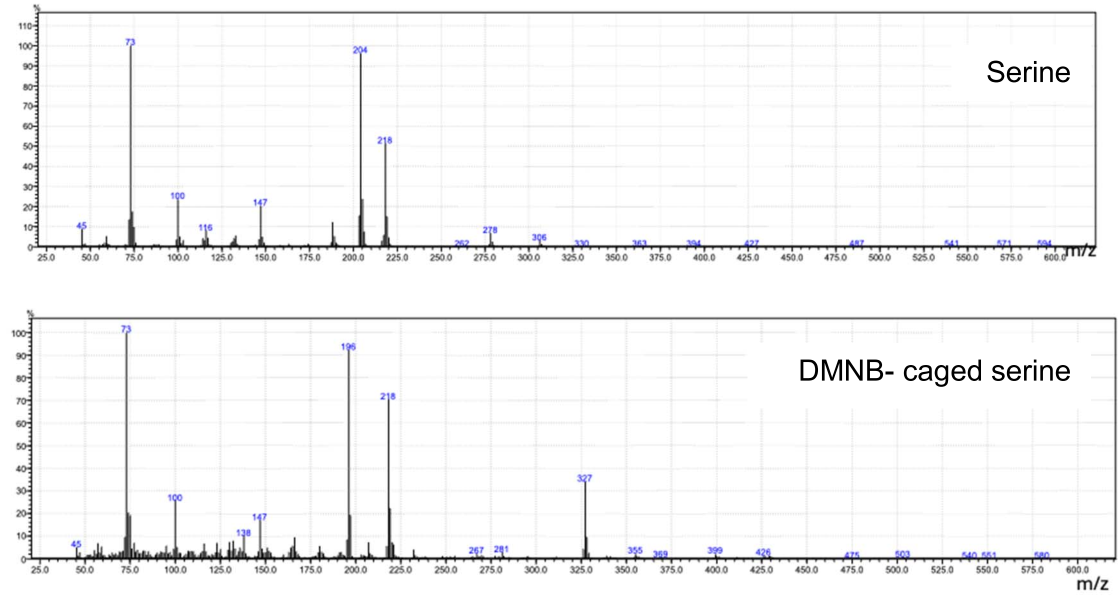

Figure S4 GCxGC-MS fragmentation spectra of the standards. Mass spectra of the standards molecules serine (m/z = 204 used as diagnostic ion, upper panel) and DMNB-caged serine (m/z = 327 as diagnostic ion, lower panel).

Supplementary Video 1

On-grid UV-Vis spectroscopy coupled with a Speed Blot in action. An example on preforming on-grid kinetic series of light-induced reaction before plunge freezing. The setup is the same as shown in Figure 3 and sections 2.4. As a note, we have not used the plunge freezing part shown in this video for the on-grid UV-Vis spectroscopy.
